# Supplementary material for: T Cell-Intrinsic Vitamin A Metabolism and Its Signaling Are Targets for Memory T Cell-Based Cancer Immunotherapy
Source: Front Immunol. 2022 Jun 30;13:935465. doi: 10.3389/fimmu.2022.935465 (PMC9280205; doi:10.3389/fimmu.2022.935465)
Supplement: Supplementary file 1 [file DataSheet_1.docx]

Supplementary Material

# Supplementary Methods

*Housing conditions*

Mice were housed in cages (3 or 5 mice per cage), which complied with the Institute of Laboratory Animals Research (ILAR) guidelines, and maintained on MF diet (Oriental Yeast Co., Tokyo, Japan). For the experiments in the infection model, gamma-ray-irradiated CRF-1 diet (Oriental Yeast Co.) were used, instead of MF diet. The animal room had a controlled 12/12-h light/dark cycle (lights on at 8:00 AM), temperature (23 ± 1.5°C), and humidity (45% ± 15%). Mice such as ones purchased from provider and moved from other rooms were provided for experiments after about 1 week of acclimatisation period.

*Microarray*

P1, P2, and P4 cells (0.17–1.69 × 10^7^ cells) were sorted from a resting CD4^+^ T-cell clone and RNA was extracted as described above. RNA quality was assessed using the 2100 Bioanalyzer (Agilent Technologies, Santa Clara, CA, USA). A microarray analysis was performed using the Affymetrix GeneChip HG-U133 Plus 2.0 (Affymetrix, Santa Clara, CA, USA) by TaKaRa Bio Inc. Genes were extracted according to the following criteria: 1 ≦ log2 ratio and detection call “Present” in the Experimental sample; -1 ≧ log2 ratio and detection call “Present” in the Base sample. All comparisons fit the two conditions. The extracted genes are listed in Supplementary Table 1.

*T-cell assessments using Rdh10-lacZ reporter mouse*

For the detection of Rdh10 expression in T cells and the evaluation of memory formation in Rdh10^hi^ or Rdh10^lo^ T cells, OT-I cells were isolated from the spleen of naïve *Rdh10^lacZ/wt^ Rag1^-/-^* OT-I CD45.1^+^ mice (Rdh10-lacZ reporter mice) and transferred into sex-matched C57BL/6J (CD45.2^+^) mice (1 × 10^5^ per mouse). On the following day, the mice were infected with LM-OVA and then used to detect Rdh10 (lacZ) expression in T cells. For the adoptive transfer of Rdh10^hi^ or Rdh10^lo^ OT-I cells, the transferred OT-I cells were magnetically enriched using biotin-conjugated anti-CD45.1 mAb and streptavidin-conjugated magnetic beads. The enriched cells were stained with fluorescein di-β-d-galactopyranoside (FDG) using the FluoReporter lacZ Flow Cytometry Kit (Thermo Fisher Scientific, San Jose, CA, USA), followed by staining of CD3, CD45.1, and CD45.2. Rdh10^hi^ and Rdh10^lo^ OT-I cells were sorted from the CD3^+^CD45.1^+^CD45.2^-^ fraction and then individually transferred into LM-OVA-infected B6 mice (1 × 10^5^ per mouse). At memory phase, mice were examined for frequencies in the blood of the transferred OT-I cells by flow cytometry and re-challenged with LM-OVA. Five days later, their frequencies were measured again in the blood.

*In vivo functional assay of Rdh10-overexpressed OT-I cells*

CD8^+^ T cells were isolated from splenocytes of naïve OT-I Rag^-/-^ mice, stimulated with irradiated and SIINFEKL peptide-pulsed splenocytes for 20 h, and then spin-infected with a retrovirus encoding mouse Rdh10 or mock control. Twelve hours later, the cells were washed and plated for 48 h in complete medium supplemented with IL-7 (5 ng/ml) and IL-15 (10 ng/ml). GFP^+^ cells were sorted and transferred into wild-type mice (4 × 10^4^ cells/mouse), followed by LM-OVA infection. Subsequently, the expansion and surface phenotype of the transferred GFP^+^ cells were examined in the blood using flow cytometry.

*Treatment of Human CD8^+^ T cells with RAR agonist/antagonist*

Naïve CD8^+^ T cells from peripheral blood mononuclear cells were enriched by Human Naive CD8 T Cell Enrichment Set (BD Bioscience, Franklin Lakes, NJ, USA), stimulated with plate-bound anti-CD3 (2 μg/ml) and soluble anti-CD28 mAbs (2 μg/ml), and cultured in the presence of IL-2 (20 IU/ml) and RA (1 μM), LE540 (10 μM), or dimethyl sulfoxide (DMSO). Three days later, the cells were harvested, washed twice, and further cultured for 4 days in 20 IU/ml of IL-2-supplemented medium. Subsequently, the cells were examined for CD62L expression by flow cytometry or labeled with CTV (1.5 μM) and cultured to investigate proliferative capacity as described elsewhere.

# Supplementary Figures and Tables

## Supplementary Figures


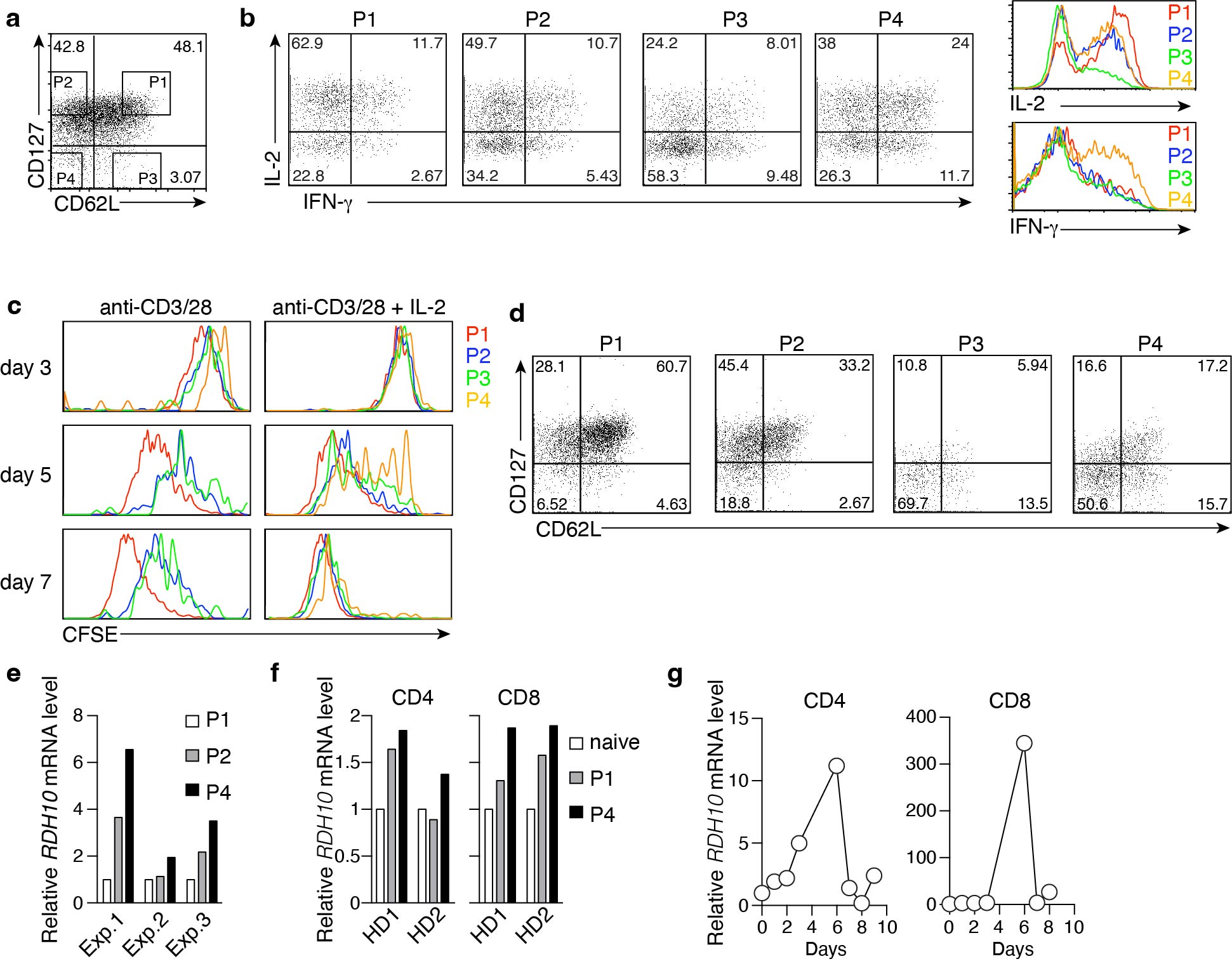


**Supplementary Figure 1.** **Identification of RDH10 associated with effector T cells.** **a** Representative dot plot showing CD62L and CD127 expression in a WT1-specific CD4^+^ T cell clone. **b**-**e** P1–4 populations indicated in **a** were sorted and used for the experiments described below. **b** Representative dot plots and histograms showing cytokine production in each population after 4 h of PMA/Ionomycin stimulation. **c** The T cells were labeled with CFSE, cultured under the indicated conditions, and evaluated for their proliferation capacity. **d** Representative dot plots showing CD62L and CD127 expression after 8 days of culture with antigenic peptide (WT1_332_)-pulsed dendritic cells. Representative data from three independent experiments are shown (**a**-**d**). **e** *RDH10* mRNA expression levels in each population. **f** *RDH10* mRNA expression levels in each population from healthy donors (HDs). Each population was sorted from CD4^+^ and CD8^+^ T cells of PBMCs and measured for *RDH10* mRNA expression levels. Naïve, CD45RO^-^ CD62L^+^ CD127^+^ T cells; P1, CD45RO^+^ CD62L^+^ CD127^+^ T cells; P4, CD45RO^+^ CD62L^-^ CD127^-^ T cells. **g** Naïve CD4^+^ and CD8^+^ T cells from HD were cultured in the presence of anti-CD3, -CD28 mAb and IL-2. At the indicated time points, *RDH10* mRNA expression levels were measured. Representative data from two independent experiments are shown.


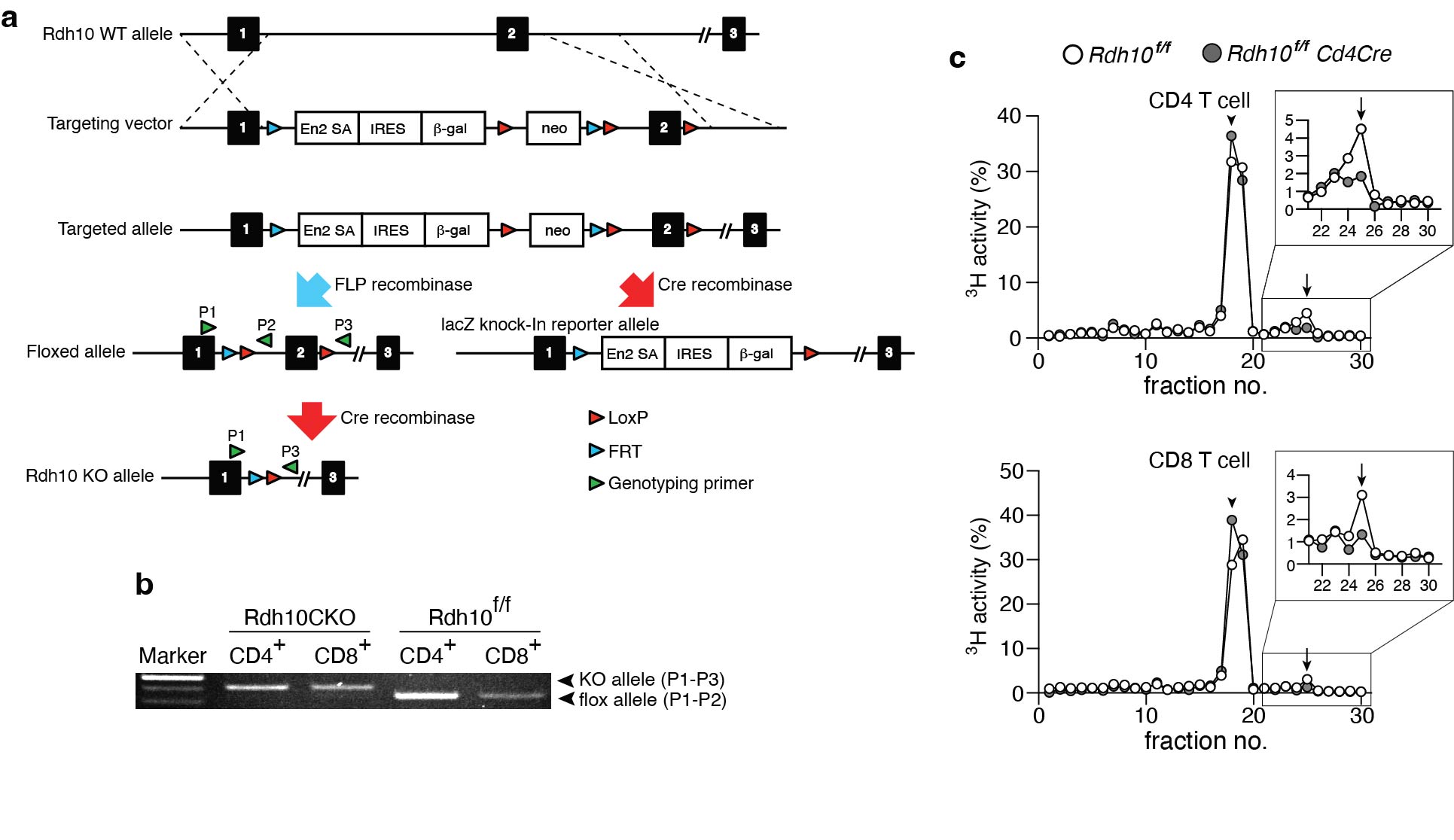


**Supplementary Figure 2. Generation of T cell-specific Rdh10 knockout mice. a** Schematic of the generation of T cell-specific Rdh10CKO and Rdh10-lacZ reporter mice. **b** Agarose gel shows KO and floxed bands obtained from genotyping using the primers as indicated in **a**. **c** RAL production from ROL was evaluated in Rdh10CKO and control T cells, as described in Fig. 1. CD4^+^ and CD8^+^ T cells were isolated from splenocytes by sorting, expanded for 12 days in the presence of anti-CD3/CD28 mAbs and IL-2, and used for the experiments. The arrowhead and arrow indicate the peak fraction of standard all-trans ROL and RAL, respectively. Representative data from three independent experiments are shown.


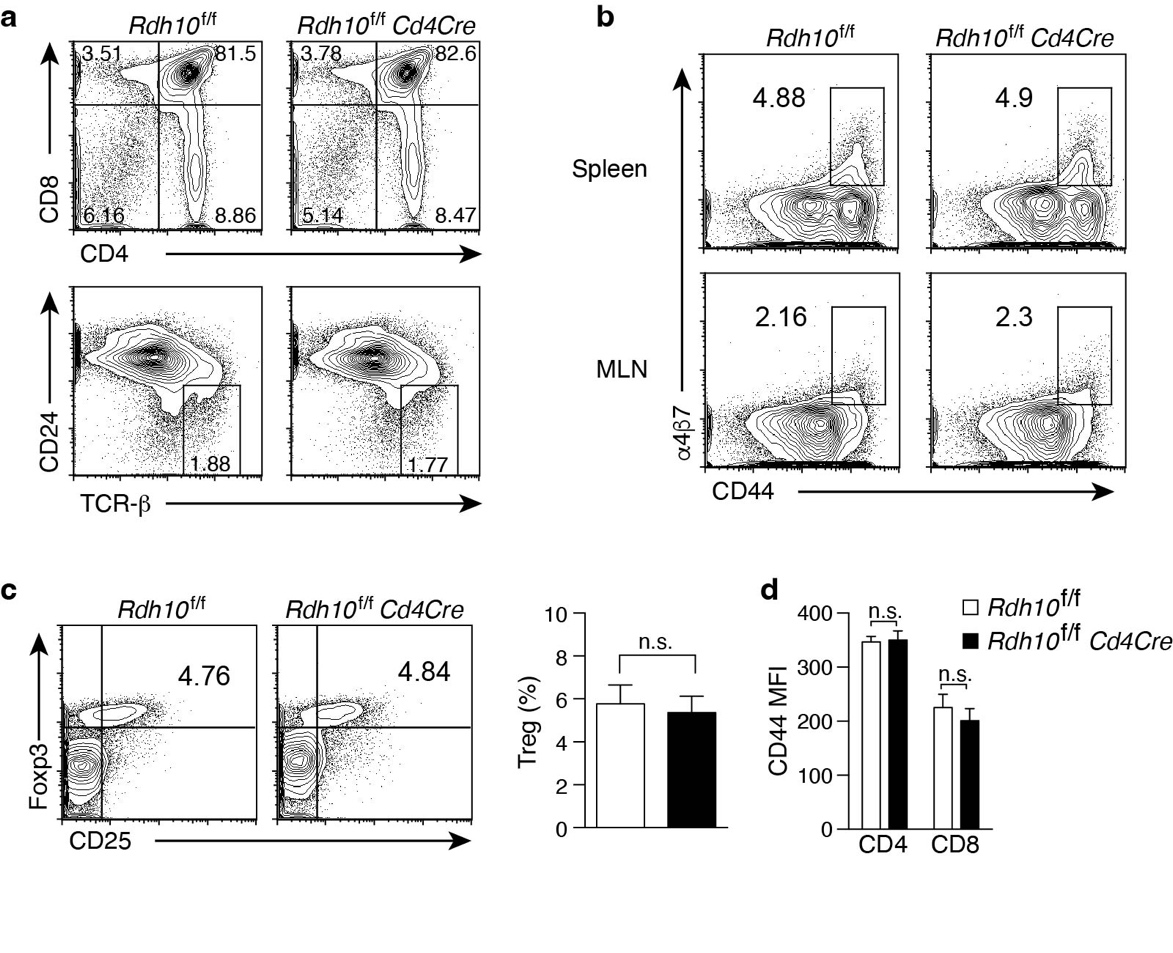


**Supplementary Figure 3. No effect of the loss of Rdh10 on T cell development and Treg and α4β7^+^ T cell induction. a** Representative dot plots showing the expression of CD4 and CD8 (*upper*), and TCR-β and CD24 (*lower*) on thymocytes from 4- to 5-week-old mice. **b** Representative dot plots showing the expression of CD44 and α4β7 integrin on CD4^+^ T cells from the spleen and MLN of 6-week-old mice. **c** Frequency of Treg in the MLN from 6- to 8-week-old mice. *left*, Representative dot plots. *right*, Graphs represent n = 8 control and n = 5 *Cd4Cre* mice. **d** CD44 expression on T cells from splenocytes of 6- to 8-week-old mice. Graphs represent n = 7 littermate control and n = 9 *Cd4Cre* mice. Data were analyzed by unpaired *t*-tests (**c** and **d**). Error bars show s.e.m. n.s., not significant.


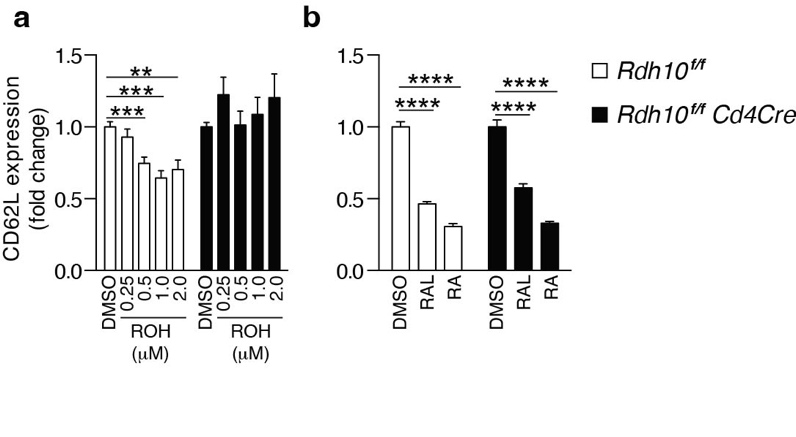


**Supplementary Figure 4. Vitamin A metabolism by Rdh10 regulates CD62L expression in T cells. a** and **b** OT-I cells isolated from Rdh10CKO and control mice were stimulated with anti-CD3/CD28 mAbs and IL-2 in the presence of ROL, RAL (1 μM), or RA (1 μM). Four days later, CD62L expression in T cells was measured by flow cytometry. Data were obtained from three (**a**) or two (**b**) independent experiments. Data were analyzed by unpaired *t*-tests. Error bars show s.e.m. **p < 0.01; ***p < 0.001; ****p < 0.0001.


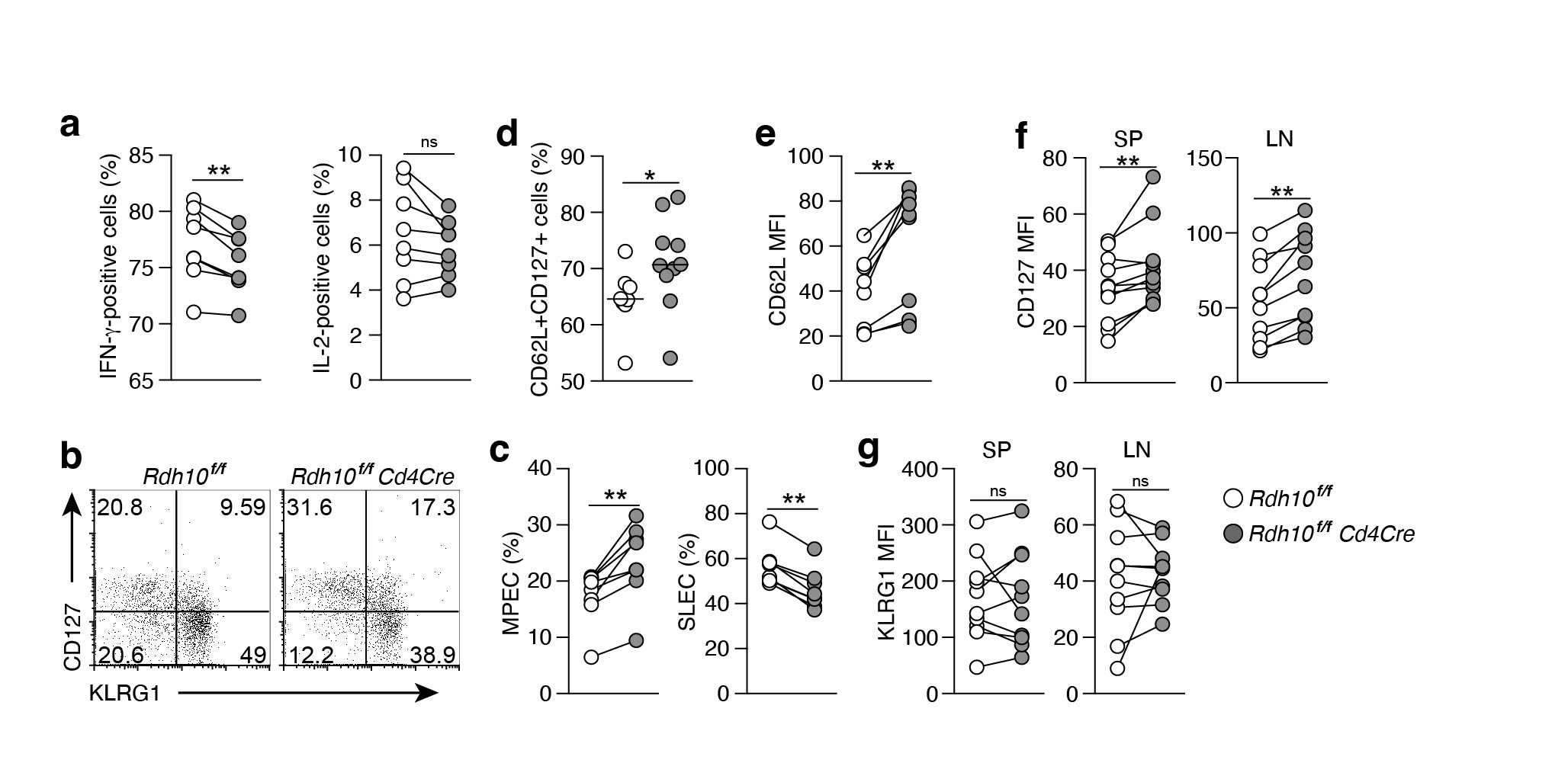


**Supplementary Figure 5. Flow cytometric analysis of Rdh10-deficient OT-I cells in an LM-OVA infection model. a**-**g** Flow cytometric analysis associated with Fig. 3. **a** Frequencies of IFN-γ (*left*) and IL-2 (*right*) -producing cells. Splenic OT-I cells from mice on day 7 post-infection (pi) were stimulated with SIINFEKL peptide for 4 h, and analyzed. **b** and **c** Frequencies of MPEC and SLEC in the spleen on day 10 pi. **d** and **e** Frequency of CD62L^+^ CD127^+^ T cells (**d**) and expression levels of CD62L (**e**) in OT-I cells in the memory phase (>30 days pi) in the lymph node. Data represent n = 8 control and n = 10 Rdh10CKO OT-I cells (**d**), and n = 8 pairs (**e**). **f** and **g** Expression of CD127 (**f**) and KLRG1 (**g**) in OT-I cells in the memory phase (>30 days pi) in the spleen and lymph node. Data represent n = 10 pairs. Data were analyzed by Wilcoxon test (**a**, **c** and **e**-**g**) or Mann–Whitney tests (**d**). *p < 0.05; **p < 0.01.

**
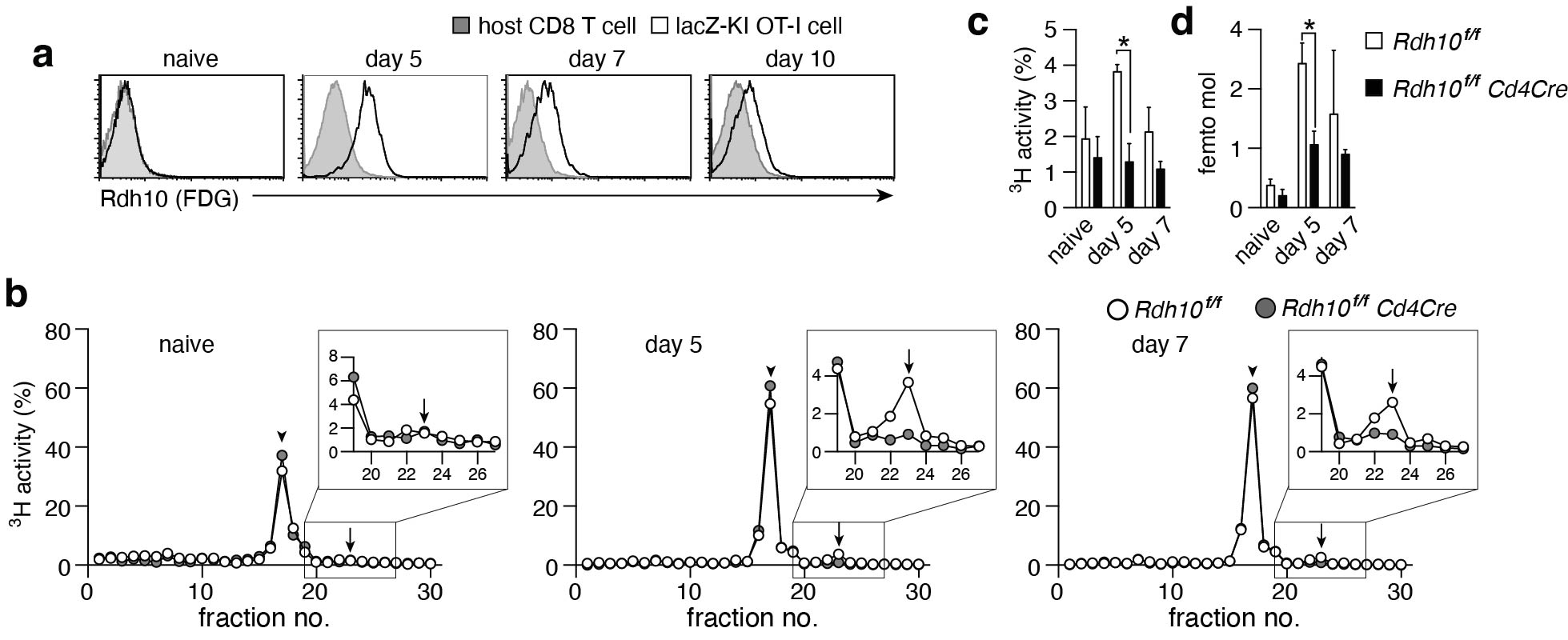
**

**Supplementary Figure 6. Transient Rdh10 induction and the resultant RAL production in activated T cells. a** Naïve OT-I cells from heterozygous Rdh10-lacZ knock-in reporter mice were intravenously transferred into B6 mice. On the following day, the mice were infected with LM-OVA via the tail vein. At the indicated time points after infection, lacZ (Rdh10) expression in OT-I cells was evaluated by FDG-staining in the spleen. Representative histograms are shown. **b**–**d** OT-I cells from Rdh10CKO and control mice were stimulated with anti-CD3/CD28 mAbs and cultured in the presence of IL-2. At the indicated days, the capacity for RAL production in OT-I cells was measured. **b** Representative data from two independent experiments are shown. Arrowhead and arrow indicate the peak fraction of standard all-trans ROL and RAL, respectively. The ^3^H activity (**c**) and absolute amount (**d**) of RAL are shown. Data from two independent experiments were analyzed by unpaired *t*-tests. Error bars show s.e.m. *p < 0.05.

**
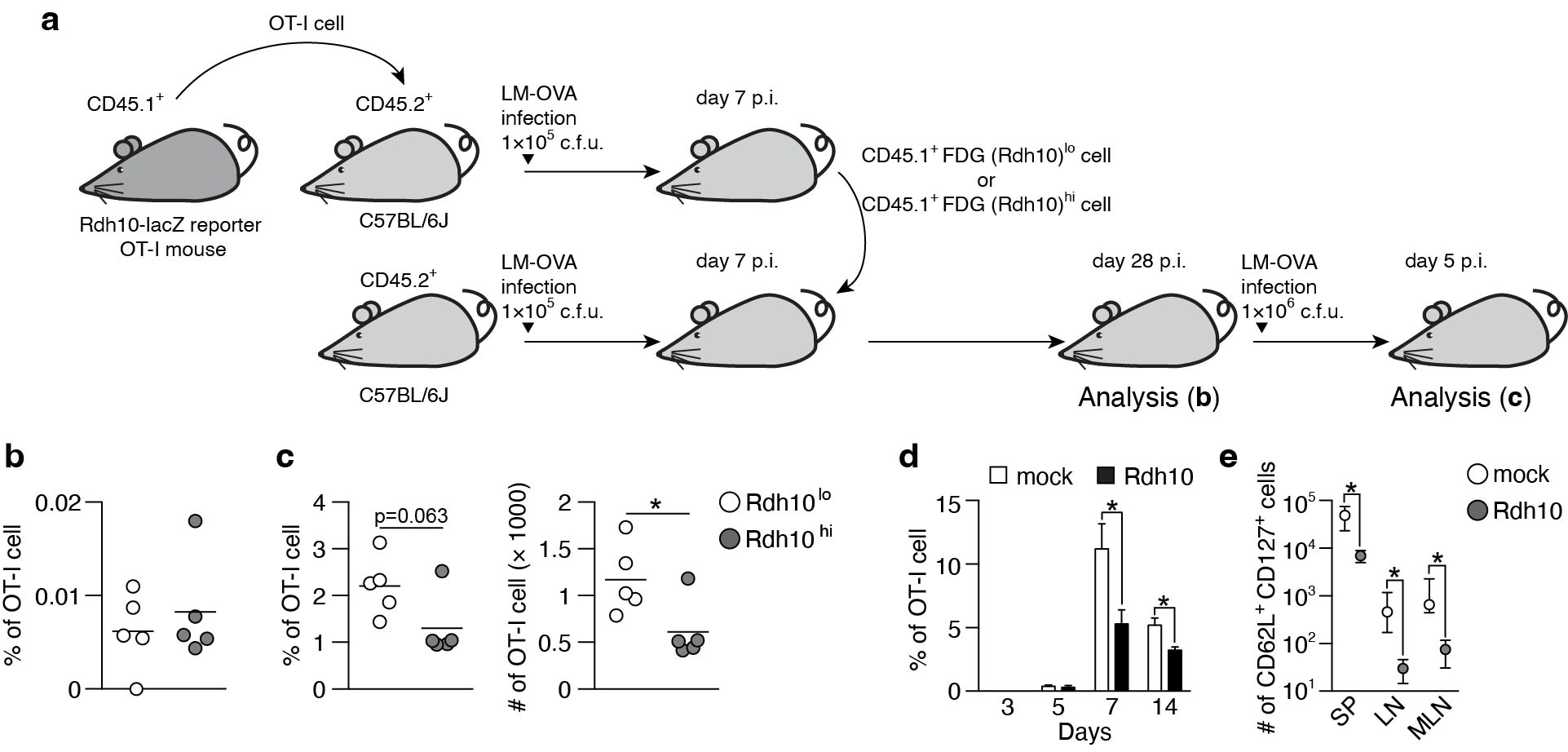
**

**Supplementary Figure 7. Rdh10 expression at the effector phase dictates the strength of the recall response. a** Experimental schematic. **b** Frequency of OT-I cells in the blood on day 28 pi. **c** Frequency (*left*) and estimated number (*right*) of OT-I cells in the blood on day 5 after LM-OVA re-challenge. **d** and **e** Freshly isolated OT-I cells were stimulated with SIINFEKL peptide-pulsed splenocytes for 20 h and then retrovirally transduced with Rdh10 or control vector (mock). The transduced OT-I cells were transferred into B6 mice and, on the following day, the mice were infected with LM-OVA. **d** Frequency of OT-I cells at the indicated time points pi. **e** Absolute number of CD62L^+^ CD127^+^ T cells in OT-I cells on day 14 pi. Data represent n=4 Rdh10-overexpressed and n=4 control OT-I cells (**d** and **e**). Data were analyzed by unpaired *t*-tests (**c** and **d**) or Mann–Whitney tests (**e**). *p < 0.05.


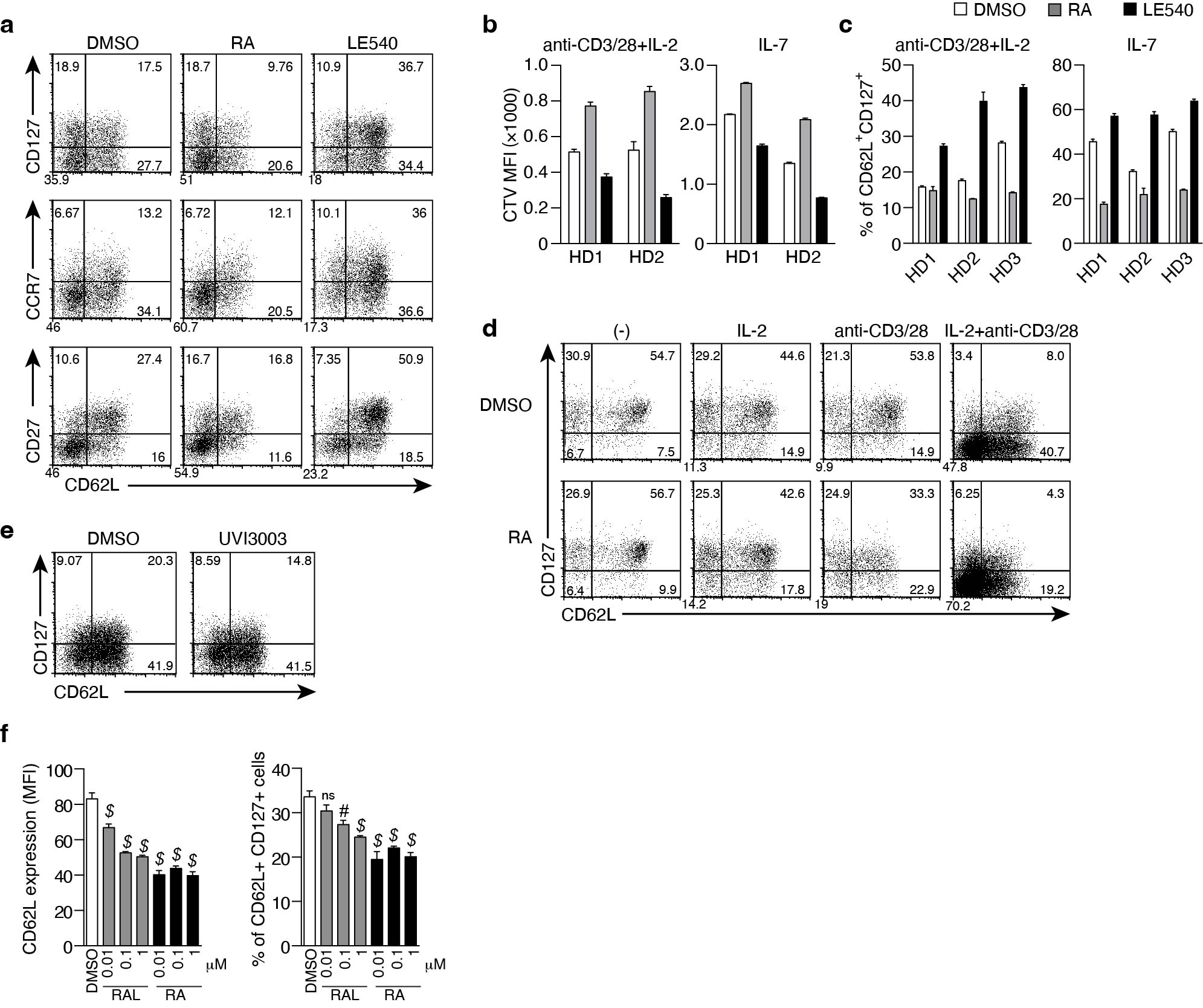


**Supplementary Figure 8. RAR signaling accelerates effector T cell differentiation.** CD45RO^+^ CD4^+^ T cells were treated with DMSO, RA, or LE540 as described in Fig. 4 and then the treated T cells were used for the following experiments (**a**–**c**). **a** Representative dot plots showing expression of CD62L, CD127, CCR7, and CD27. **b** and **c** T cells were labeled (**b**) or unlabeled (**c**) with CellTrace Violet (CTV), cultured for 4 or 6 days, respectively, under the indicated conditions, and analyzed for cell proliferation (**b**) and reconstitution capacity of T_CM_ (**c**) by flow cytometry. **d** Representative dot plots showing CD62L and CD127 expression. CD45RO^+^ CD4^+^ T cells were cultured under the indicated conditions for 7 days and analyzed. **e** Representative dot plots of the T cells treated with a pan-RXR antagonist, UVI3003, as shown in Fig. 4. **f** CD45RO^+^ CD4^+^ T cells were treated with either RAL or RA at the indicated concentrations. CD62L expression and frequencies of T_CM_ (CD62L^+^ CD127^+^ cells) were determined by flow cytometry. One-way ANOVA with post-hoc Dunnett’s test was used to compare each treatment to control (DMSO). Error bars, s.em. *^#^*p < 0.001; *^$^*p < 0.0001.


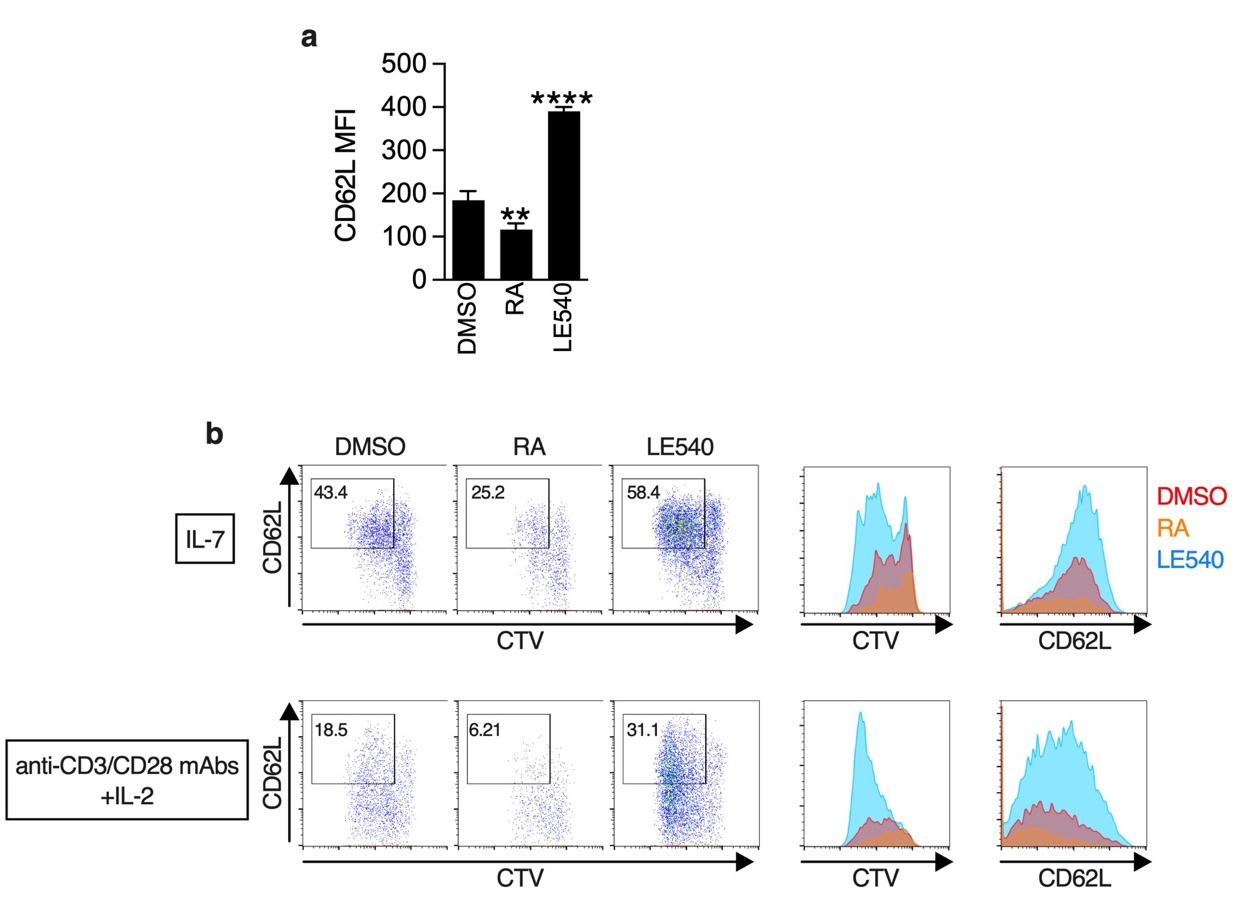


**Supplementary Figure 9. RAR signaling weakens CD62L expression and proliferative capacity in human CD8^+^ T cells.** Naive human CD8^+^ T cells were treated with DMSO, RA, or LE540 and then the treated T cells were used for the following experiments. **a** CD62L expression level in the treated T cells. Bars show mean value with SD from triplicate wells. Representative data from two independent experiments are shown. One-way ANOVA with post-hoc Dunnett’s test was used to compare each treatment to control (DMSO). **p < 0.01; ****p < 0.0001. **b** T cells were labeled with CTV, cultured for 3 days under the indicated conditions, and analyzed for cell proliferation and CD62L expression by flow cytometry. The gated cells show CD62L^+^ dividing cells that are indicator of existence of central memory T cells. Representative dot plots and histograms from two independent experiments are shown.


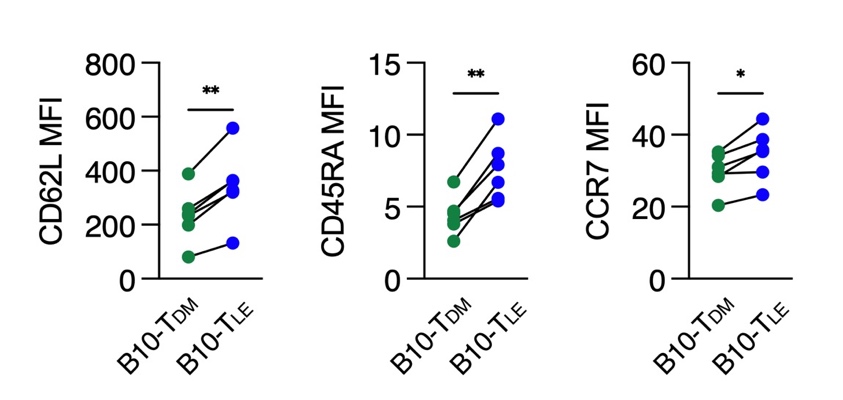


**Supplementary Figure 10. Higher expression of CD62L, CD45RA, and CCR7 in B10-T_LE_ cells.** B10-TDM and B10-TLE cells were examined for CD62L, CD45RA, and CCR7 expression level by flow cytometry before adoptive T-cell transfer. The data are from six independent experiments. Paired t-test. *p < 0.05; **p < 0.01.


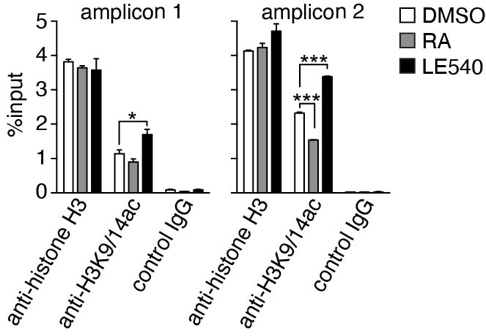


**Supplementary Figure 11. Regulation of the acetylation of histone H3 at the *CD62L* promoter by RAR signaling.** ChIP assays of human CD45RO^+^ T cells treated with DMSO, RA, or LE540 for 7 days as described in Fig 4. Data were obtained from two independent experiments. Data were analyzed by unpaired *t*-tests. Error bars show s.e.m. *p < 0.05; ***p < 0.001.


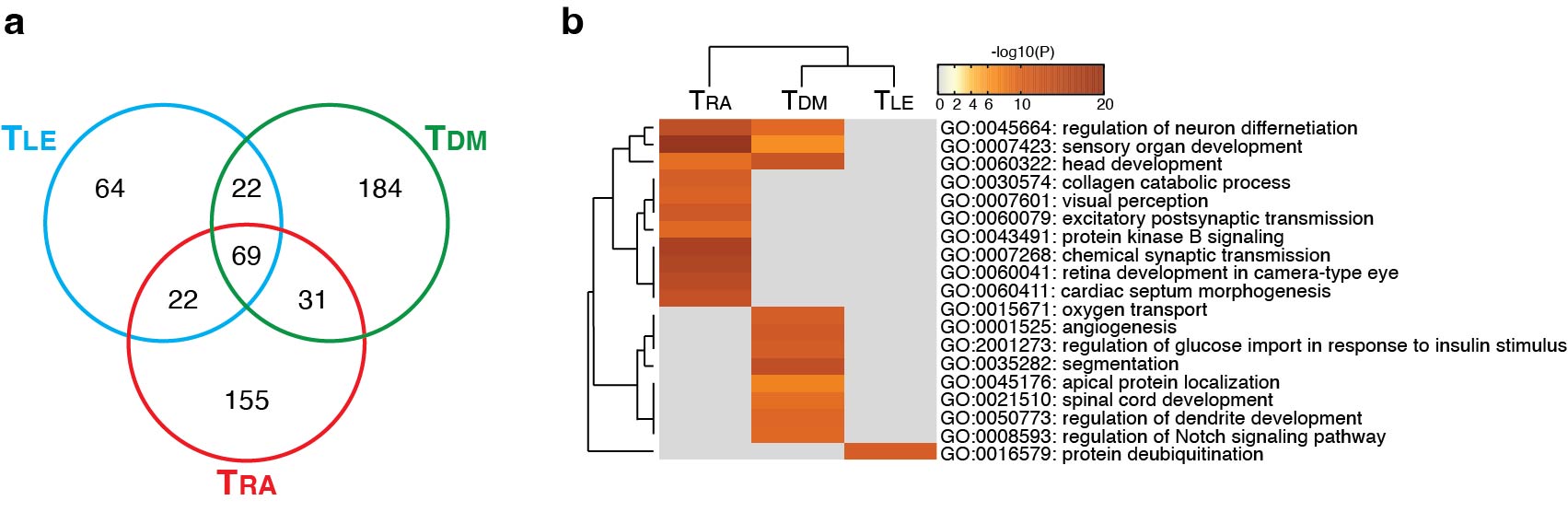


**Supplementary Figure 12. Metascape analysis of uniquely H3K27me3-oocupied genes among T_DM_, T_LE_, and T_RA_ cells.** ChIP-seq analysis was performed as described in Fig. 7. **a** Venn diagram shows the number of genes located within ±10 kb of H3K27me3-occupied regions. **b** Metascape analysis was performed on the uniquely H3K27me3-occupied genes. Pathway and Process Enrichment analysis were performed using default settings without Reactome Gene Sets.

## Supplementary Tables

| **Table S1: Different gene expression among the three populations** | |  |  |  |  |
| --- | --- | --- | --- | --- | --- |
| Probe Set ID | Gene Title | Gene Symbol | Change (Log2 Ratio) | | |
|  |  |  | P4/P1 | P2/P1 | P4/P2 |
| 232706_s_at | TraB domain containing | TRABD | **3.57** | **2.42** | **1.15** |
| 223693_s_at | Rap GTPase interactor | RADIL | **3.52** | **2.33** | **1.19** |
| 203505_at | ATP-binding cassette, sub-family A (ABC1), member 1 | ABCA1 | **3.28** | **1.04** | **2.24** |
| 1553998_at | DMRT-like family C1 /// similar to doublesex and mab-3 related transcription factor 8.1 isoform a | DMRTC1 /// LOC728656 | **3.24** | **4.98** | -1.74 |
| 1552378_s_at | retinol dehydrogenase 10 (all-trans) | RDH10 | **3.15** | **1.93** | **1.21** |
| 227467_at | retinol dehydrogenase 10 (all-trans) | RDH10 | **2.95** | **1.70** | **1.25** |
| 214161_at | Oxidative stress induced growth inhibitor family member 2 | OSGIN2 | **2.63** | **1.60** | **1.03** |
| 226021_at | retinol dehydrogenase 10 (all-trans) | RDH10 | **2.34** | **1.31** | **1.03** |
| 220397_at | Mdm1 nuclear protein homolog (mouse) | MDM1 | **2.07** | **1.00** | **1.06** |
| 214081_at | plexin domain containing 1 | PLXDC1 | **1.72** | **2.82** | -1.09 |
| 215094_at | coiled-coil domain containing 52 | CCDC52 | **1.36** | **2.38** | -1.02 |
| 214567_s_at | chemokine (C motif) ligand 1 /// chemokine (C motif) ligand 2 | XCL1 /// XCL2 | **1.25** | **3.67** | -2.42 |
| 206366_x_at | chemokine (C motif) ligand 1 | XCL1 | **1.17** | **3.03** | -1.86 |
| 239312_at | --- | --- | -1.08 | -2.34 | **1.26** |
| 1568887_at | CDNA clone IMAGE:4822266 | --- | -1.19 | -2.22 | **1.03** |
| 1552783_at | zinc finger protein 417 | ZNF417 | -1.37 | -2.76 | **1.40** |
| 214710_s_at | cyclin B1 | CCNB1 | -2.25 | -1.22 | -1.03 |
| 204315_s_at | G-2 and S-phase expressed 1 | GTSE1 | -2.31 | -1.19 | -1.12 |
| 201291_s_at | topoisomerase (DNA) II alpha 170kDa | TOP2A | -2.34 | -1.04 | -1.30 |
| 202870_s_at | cell division cycle 20 homolog (S. cerevisiae) | CDC20 | -2.37 | -1.17 | -1.20 |
| 202705_at | cyclin B2 | CCNB2 | -2.44 | -1.22 | -1.22 |
| 202580_x_at | forkhead box M1 | FOXM1 | -2.45 | -1.31 | -1.14 |
| 207165_at | hyaluronan-mediated motility receptor (RHAMM) | HMMR | -2.58 | -1.54 | -1.04 |
| 1566265_at | Full length insert cDNA YQ80D07 | --- | -2.99 | -1.19 | -1.80 |
| 228729_at | cyclin B1 | CCNB1 | -3.35 | -1.16 | -2.19 |
| 1560901_at | CDNA FLJ38467 fis, clone FEBRA2021636 | --- | -3.42 | -1.06 | -2.35 |
| P1, P2 and P4 represent CD62L+CD127+, CD62L-CD127+, CD62L-CD127- cells, respectively. | |  |  |  |  |

| **Supplementary Table 2. List of Opend Genes** | | |  |  |  |  |
| --- | --- | --- | --- | --- | --- | --- |
| *Opend genes determined by H3K9/14ac* | | |  | *Opend genes determined by H3K27me3* | | |
|  | *Official Gene Symbol* |  |  | *Official Gene Symbol* | | |
| TDM vs TLE | TRA vs TDM | TRA vs TLE |  | TDM vs TLE | TRA vs TDM | TRA vs TLE |
| C2CD3 | CCR9 | SMG6 |  | SLC24A3 | MIR3198-1 | SNAR-A6 |
| TOPORS-AS1 | WDR31 | CD38 |  | PDE11A | SHROOM2 | CAMTA1 |
| ZNF154 | LOC101927156 | CCR9 |  | REXO1L2P | SLC6A20 | SNAR-A4 |
| SMG6 | FYCO1 | FYCO1 |  | FRG2 | NBPF25P | SLC6A20 |
| MYLIP | SSC5D | LOC101927156 |  | LINGO1 | ZP3 | LOC284294 |
| GFI1 | LZTFL1 | LZTFL1 |  | GRM8 | IPCEF1 | SNAR-A8 |
| LINC00582 | PLEKHA7 | ARHGEF18 |  | PCBP3 | EBF4 | LOC100506990 |
| C1GALT1 | ATAD3B | PHF21A |  | CCDC144A | NCR3LG1 | SNAR-A7 |
| RPL37 | ATAD3C | MCCC1 |  |  | FRG1 | SNAR-D |
| LOC101927156 | KCTD1 | C1orf186 |  |  | OPRM1 | SNAR-A5 |
| TSNAX-DISC1 | COLGALT1 | ABTB2 |  |  | NCAM2 | SNAR-B1 |
| NDUFB6 | UNC13A | EVI5 |  |  | LZTFL1 | SNAR-A9 |
| LGALS12 | MED13L | ABCA1 |  |  | BID | SNAR-A11 |
| UCP3 |  | COLGALT1 |  |  |  | TMEM242 |
| CARD6 |  | RCBTB1 |  |  |  | ACTG2 |
| TOPORS |  | ABCC1 |  |  |  | CLDN11 |
| ABTB2 |  | UNC13A |  |  |  | LZTFL1 |
| SMURF2 |  | SLC26A4-AS1 |  |  |  | LINC00305 |
| SNORD72 |  | SLC26A4 |  |  |  | ASTN2 |
| BTRC |  |  |  |  |  | MIR6828 |
|  |  |  |  |  |  | GNG4 |
|  |  |  |  |  |  | SLC26A4-AS1 |
|  |  |  |  |  |  | MIR5096 |
|  |  |  |  |  |  | CEACAM22P |
|  |  |  |  |  |  | SNAR-B2 |
|  |  |  |  |  |  | GET4 |
|  |  |  |  |  |  | SLC26A4 |
| **Supplementary Table 2. *Continued*** | |  |  |  |  |  |
|  |  |  |  |  |  | SNAR-A10 |
|  |  |  |  |  |  | LOC100420587 |
|  |  |  |  |  |  | DLEU2 |
|  |  |  |  |  |  | MDGA2 |
|  |  |  |  |  |  | SLC6A11 |
|  |  |  |  |  |  | SNAR-A14 |
|  |  |  |  |  |  | LINGO1 |
|  |  |  |  |  |  | G6PC |
|  |  |  |  |  |  | DPF3 |
|  |  |  |  |  |  | DLEU1 |
|  |  |  |  |  |  | C1orf127 |
|  |  |  |  |  |  | LINC00671 |
|  |  |  |  |  |  | ABTB2 |
|  |  |  |  |  |  | MIAT |
|  |  |  |  |  |  | SNAR-A3 |
|  |  |  |  |  |  | XKR4 |
|  |  |  |  |  |  | ADAP1 |
|  |  |  |  |  |  | PDE4B |

| **Supplementary Table 3. List of Closed Genes** | | |  |  |  |  |
| --- | --- | --- | --- | --- | --- | --- |
| *Closed genes determined by H3K9/14ac* | | |  | *Closed genes determined by H3K27me3* | | |
| *Official Gene Symbol* | | |  | *Official Gene Symbol* | | |
| TDM vs TLE | TRA vs TDM | TRA vs TLE |  | TDM vs TLE | TRA vs TDM | TRA vs TLE |
| LINC00684 | C2CD3 | SYNJ2BP-COX16 |  | DLG5-AS1 | KCNAB1 | PCAT19 |
| FOXO3B | PGK1 | PLA2G4E-AS1 |  | SCML4 | SHKBP1 | HMSD |
| FAM226B | ENTPD3-AS1 | RCBTB2 |  | DLG5 | LTBP4 | SMOC1 |
| RNF216-IT1 | SAMSN1 | LINC00684 |  | FGF14-IT1 | CABLES1 | DLG5-AS1 |
| FAM226A | PHF20 | MIR1268A |  | DNAH8 | LRRC15 | DLG5 |
| PDIA4 | MCCC2 | PPM1G |  | FGF14-AS2 | CPN2 | NEUROG2 |
| FTSJ1 | ZNF789 | MIR6891 |  | ID1 |  | HORMAD2 |
| ABCA2 | LINC00892 | MB21D2 |  | DDX11L16 |  | CERKL |
| RPLP2 | GNG13 | ATR |  | ASAP1 |  | PELI2 |
| LOC101928402 | HBP1 | TGFBR3 |  | C17orf105 |  | ASAP1 |
| SNORA52 | SPATA33 | TMCO1 |  | PLEKHH3 |  | TCF7 |
| POMT1 | MIR484 | DSTNP2 |  | LOC101926913 |  | CLUL1 |
| STAG2 | BAGE | ZNF789 |  | GUCY2D |  | TMCC2 |
| UCK1 | FANCC | NOLC1 |  | CDON |  | LENG8 |
| NBPF25P | IQGAP1 | MSC |  | FERMT2 |  | LOC101926913 |
| NOL4L | BAGE2 | SNHG5 |  | JAZF1 |  | KIT |
| SARS | BAGE3 | LAMTOR1 |  | ADRA1B |  | KCNAB1 |
| RNF216 | BAGE4 | MIR3146 |  | GRASP |  | ENAH |
| ZNF737 | BAGE5 | DDX6 |  | CD8A |  | ELOVL7 |
| MT1M | PRR25 | IMMT |  | IPCEF1 |  | TMEM178A |
| MT1JP | MTOR | SNX17 |  | MT1M |  | RNF216 |
| DRC1 | RPL14 | ABCA2 |  | MT1JP |  | MIR5692C1 |
| MT1H | NTRK1 | COQ2 |  | MT1IP |  | MT1M |
| LOC100132304 | SH2D2A | ABL1 |  | EML1 |  | MT1JP |
| MT1IP | SNX6 | SYNJ2BP |  | MT1H |  | DPY19L2P2 |
| NPDC1 | GNAS-AS1 | C15orf39 |  | ITIH5 |  | MT1IP |
| SLC38A5 | SETBP1 | HRH2 |  | CCR10 |  | MT1H |
| **Supplementary Table 3. *Continued*** | |  |  |  |  |  |
| MT1A | ATP5J2-PTCD1 | MIR3610 |  | TLR5 |  | KDM4D |
| MT1F | BIN3-IT1 | RNF216 |  | MT1A |  | MPO |
| MT1G | DOCK9 | NTRK2 |  | MT1F |  | MT1A |
| MT1E | PRR34 | AP5S1 |  | MT1G |  | MT1F |
| C9orf139 | RASIP1 | TRAF3IP3 |  | VEPH1 |  | MT1G |
| ZNF286B | EMILIN2 | LOC100132304 |  | MT1E |  | LOC101928435 |
| INE1 | HILPDA | RPS6KA5 |  | SP5 |  | MT1E |
| TMEM55B | MAMSTR | FOXK1 |  | SLC9A3 |  | DKK3 |
| LSM14A | GFOD1 | MADCAM1 |  | RMND5A |  | ERICH2 |
| LOC101927914 | UCP3 | TIAM1 |  | HS3ST3A1 |  | WNT10A |
| PIK3CA | PLB1 | SNORD50B |  | MIR3193 |  | STARD4-AS1 |
| LOC101928739 | BTG2 | MIR3692 |  | MN1 |  | TBC1D4 |
| ASB4 | LOC441081 | LOC101928435 |  | MIR137 |  | DAGLA |
| PNPLA2 | APOC3 | GIPR |  | DUSP3 |  | COLGALT2 |
| MT1DP | LOC100289473 | LINC00423 |  | MIR4456 |  | CABLES1 |
| TIAM2 | ATP5J2 | ARHGAP15 |  | CDH13 |  | CDC42EP5 |
| APEX1 | MIR548AZ | SEC62 |  | FGF14 |  | PIK3CA |
| DHRS7B | APOA1 | BRAP |  | RBPMS-AS1 |  | CCSER1 |
| UBA1 | KCNJ1 | TNFSF14 |  | MT1DP |  | LOC101928739 |
| HLA-DRB1 | SIK3 | TMEM55B |  | FGF5 |  | SLC1A2 |
| FUT7 | LOC101927166 | LOC101927914 |  | TUBG2 |  | CECR2 |
| ARHGAP25 | GNAS | GPR55 |  | ACVR1B |  | SAMD4A |
| RBM10 | LOC102467080 | LOC100506746 |  | LINC01124 |  | CA7 |
| OSGEP | DUSP5P1 | SNRPD2 |  | PROM1 |  | LENG9 |
| PNP | ABLIM1 | RAP2A |  | MIR2682 |  | MT1DP |
| BRE | PRR34-AS1 | ZDHHC14 |  | PTX3 |  | CLTCL1 |
| LOC285766 | NDUFB6 | GOLGA7 |  | CNTNAP1 |  | IGF2BP1 |
| KIAA1324 | ATG14 | ZFYVE28 |  | SLC7A10 |  | WNT6 |
| PIDD1 | RBMS1 | RPL34-AS1 |  | OPRM1 |  | IGF2BP3 |
| ZC3H3 | INTS4 | DNAJC28 |  | CD70 |  | TNFAIP8L3 |
|  | SPATA13-AS1 | TNFAIP8 |  | GMDS |  | LPO |
| **Supplementary Table 3. *Continued*** | |  |  |  |  |  |
|  | GMFB | TWISTNB |  | RBPMS |  | NAE1 |
|  | ZNF26 | WDR47 |  | PDE8B |  | TYMSOS |
|  | OGDH | TYMSOS |  | WNT3A |  | LOC101929710 |
|  | MOB3A | LOC102467080 |  | MIR137HG |  | NREP |
|  | UBIAD1 | MCM10 |  | CAMSAP2 |  | CD70 |
|  | BIN3 | THAP9 |  |  |  | HECW2 |
|  | RASA2 | CENPO |  |  |  | AARD |
|  | LOC101928809 | EXOSC10 |  |  |  |  |
|  | SP100 | BCAT1 |  |  |  |  |
|  | GUSBP3 | GNAI2 |  |  |  |  |
|  | SPAG1 | USP11 |  |  |  |  |
|  | ITPR1 | RAD21-AS1 |  |  |  |  |
|  | HIF1A-AS1 | GNA14-AS1 |  |  |  |  |
|  | SPATA13 | XKR6 |  |  |  |  |
|  | ZFYVE21 | LINC00271 |  |  |  |  |
|  | IRAK1BP1 | GMFB |  |  |  |  |
|  | TEX21P | BCL2 |  |  |  |  |
|  | NUGGC | OGDH |  |  |  |  |
|  | DOCK9-AS2 | CRYL1 |  |  |  |  |
|  | SP3 | RB1 |  |  |  |  |
|  | TOPORS-AS1 | SEC31A |  |  |  |  |
|  | SDAD1 | TRAPPC8 |  |  |  |  |
|  | ESR2 | PIGX |  |  |  |  |
|  | SHQ1 | BTD |  |  |  |  |
|  | RPL39 | ACAD10 |  |  |  |  |
|  | PJA2 | IKZF1 |  |  |  |  |
|  | TMEM97 | ITPR1 |  |  |  |  |
|  | APOA1-AS | LOC100506804 |  |  |  |  |
|  | ELP3 | LOC101929125 |  |  |  |  |
|  | XRCC3 | B4GALT7 |  |  |  |  |
|  | LCK | TIGIT |  |  |  |  |
| **Supplementary Table 3. *Continued*** | |  |  |  |  |  |
|  | NFKB1 | SMDT1 |  |  |  |  |
|  | SEPHS1 | LRTOMT |  |  |  |  |
|  | PRKAR2B | NUGGC |  |  |  |  |
|  | LINC00899 | HPSE |  |  |  |  |
|  | NCOA2 | AP5Z1 |  |  |  |  |
|  | SMA5 | CCNL2 |  |  |  |  |
|  | LINC01136 | PTRHD1 |  |  |  |  |
|  | MACF1 | DIAPH2 |  |  |  |  |
|  | SNORA69 | PRKCQ |  |  |  |  |
|  | RERE | GPR107 |  |  |  |  |
|  | CARD11 | LINC01353 |  |  |  |  |
|  | FAM167B | MT1M |  |  |  |  |
|  | TOPORS | ZNF543 |  |  |  |  |
|  | MIR7641-2 | MT1IP |  |  |  |  |
|  | KIAA0430 | MT1H |  |  |  |  |
|  | CHTF18 | RRN3P2 |  |  |  |  |
|  | CNTRL | MT1L |  |  |  |  |
|  | IQSEC1 | PA2G4 |  |  |  |  |
|  | CHMP1A | ENTPD1-AS1 |  |  |  |  |
|  | NDE1 | ZBTB20 |  |  |  |  |
|  | IFT20 | NCK2 |  |  |  |  |
|  | RHOU | MT1F |  |  |  |  |
|  | ZDHHC20 | LINC00520 |  |  |  |  |
|  | MTMR9LP | MT1G |  |  |  |  |
|  | SNX9 | C9orf139 |  |  |  |  |
|  |  | MT1E |  |  |  |  |
|  |  | TSHZ2 |  |  |  |  |
|  |  | GJB6 |  |  |  |  |
|  |  | AK2 |  |  |  |  |
|  |  | DTWD1 |  |  |  |  |
|  |  | RGMB-AS1 |  |  |  |  |
| **Supplementary Table 3. *Continued*** | |  |  |  |  |  |
|  |  | TMEM14A |  |  |  |  |
|  |  | LINC01136 |  |  |  |  |
|  |  | LOC100147773 |  |  |  |  |
|  |  | MACF1 |  |  |  |  |
|  |  | MIR3909 |  |  |  |  |
|  |  | C17orf75 |  |  |  |  |
|  |  | FBXO21 |  |  |  |  |
|  |  | NBPF20 |  |  |  |  |
|  |  | DOPEY1 |  |  |  |  |
|  |  | ATXN7L3 |  |  |  |  |
|  |  | CALCOCO2 |  |  |  |  |
|  |  | HIST1H3A |  |  |  |  |
|  |  | EIF2B4 |  |  |  |  |
|  |  | LOC100996437 |  |  |  |  |
|  |  | LDLRAD4 |  |  |  |  |
|  |  | USP12-AS1 |  |  |  |  |
|  |  | PAK2 |  |  |  |  |
|  |  | MIR548N |  |  |  |  |
|  |  | QPCTL |  |  |  |  |
|  |  | TMEM50B |  |  |  |  |
|  |  | KRTAP16-1 |  |  |  |  |
|  |  | MFN2 |  |  |  |  |
|  |  | BATF3 |  |  |  |  |
|  |  | HIST1H4B |  |  |  |  |
|  |  | C3 |  |  |  |  |
|  |  | TIMM23B |  |  |  |  |
|  |  | CRYGN |  |  |  |  |
|  |  | HIST1H4A |  |  |  |  |
|  |  | USP28 |  |  |  |  |
|  |  | SNORD50A |  |  |  |  |
|  |  | EZH2 |  |  |  |  |
| **Supplementary Table 3. *Continued*** | |  |  |  |  |  |
|  |  | IL12RB2 |  |  |  |  |
|  |  | FAM102A |  |  |  |  |
|  |  | UBE3D |  |  |  |  |
|  |  | HLA-B |  |  |  |  |
|  |  | PBX2 |  |  |  |  |
|  |  | LOC100506274 |  |  |  |  |
|  |  | CFAP99 |  |  |  |  |
|  |  | MIR3907 |  |  |  |  |
|  |  | NFKBIZ |  |  |  |  |
|  |  | MIR6124 |  |  |  |  |
|  |  | TOM1 |  |  |  |  |
|  |  | STX5 |  |  |  |  |
|  |  | RNF216-IT1 |  |  |  |  |
|  |  | FAM226A |  |  |  |  |
|  |  | HYDIN |  |  |  |  |
|  |  | CAPZA2 |  |  |  |  |
|  |  | EHD4 |  |  |  |  |
|  |  | LOC100505478 |  |  |  |  |
|  |  | RHPN1 |  |  |  |  |
|  |  | LINC01108 |  |  |  |  |
|  |  | RPL13P5 |  |  |  |  |
|  |  | SMAD4 |  |  |  |  |
|  |  | MBP |  |  |  |  |
|  |  | FNBP1 |  |  |  |  |
|  |  | MIR6069 |  |  |  |  |
|  |  | MIR568 |  |  |  |  |
|  |  | SH3RF3 |  |  |  |  |
|  |  | TOP1MT |  |  |  |  |
|  |  | KIF1B |  |  |  |  |
|  |  | LOC148413 |  |  |  |  |
|  |  | PSTPIP1 |  |  |  |  |
| **Supplementary Table 3. *Continued*** | |  |  |  |  |  |
|  |  | SSBP2 |  |  |  |  |
|  |  | PTGER4 |  |  |  |  |
|  |  | NPDC1 |  |  |  |  |
|  |  | ATP5J2-PTCD1 |  |  |  |  |
|  |  | LOC100505530 |  |  |  |  |
|  |  | ANK3 |  |  |  |  |
|  |  | RGMB |  |  |  |  |
|  |  | SPO11 |  |  |  |  |
|  |  | THAP9-AS1 |  |  |  |  |
|  |  | LOC730101 |  |  |  |  |
|  |  | TMED9 |  |  |  |  |
|  |  | CREBBP |  |  |  |  |
|  |  | COX16 |  |  |  |  |
|  |  | PPRC1 |  |  |  |  |
|  |  | NAGA |  |  |  |  |
|  |  | LOC100128164 |  |  |  |  |
|  |  | LY9 |  |  |  |  |
|  |  | NARS |  |  |  |  |
|  |  | SDHA |  |  |  |  |
|  |  | FBXO32 |  |  |  |  |
|  |  | DUSP4 |  |  |  |  |
|  |  | GPR65 |  |  |  |  |
|  |  | TXNIP |  |  |  |  |
|  |  | OSBPL6 |  |  |  |  |
|  |  | ACOT9 |  |  |  |  |
|  |  | ATP5J2 |  |  |  |  |
|  |  | CUL4A |  |  |  |  |
|  |  | HBEGF |  |  |  |  |
|  |  | OSBPL10 |  |  |  |  |
|  |  | MIR632 |  |  |  |  |
|  |  | IGF2BP3 |  |  |  |  |
| **Supplementary Table 3. *Continued*** | |  |  |  |  |  |
|  |  | DENND4C |  |  |  |  |
|  |  | NDUFA6 |  |  |  |  |
|  |  | HACL1 |  |  |  |  |
|  |  | APEX1 |  |  |  |  |
|  |  | KRTAP17-1 |  |  |  |  |
|  |  | PGAP1 |  |  |  |  |
|  |  | LOC100505549 |  |  |  |  |
|  |  | CD226 |  |  |  |  |
|  |  | RANBP3 |  |  |  |  |
|  |  | CLCC1 |  |  |  |  |
|  |  | FAM109B |  |  |  |  |
|  |  | ADTRP |  |  |  |  |
|  |  | DOCK10 |  |  |  |  |
|  |  | C10orf88 |  |  |  |  |
|  |  | FUT7 |  |  |  |  |
|  |  | MRPL35 |  |  |  |  |
|  |  | CDK16 |  |  |  |  |
|  |  | RAE1 |  |  |  |  |
|  |  | TYMS |  |  |  |  |
|  |  | FAM227B |  |  |  |  |
|  |  | HECW2 |  |  |  |  |
|  |  | OSGEP |  |  |  |  |
|  |  | BRE |  |  |  |  |
|  |  | WDR74 |  |  |  |  |
|  |  | HIST1H1A |  |  |  |  |
|  |  | LOC285766 |  |  |  |  |
|  |  | NDUFV2 |  |  |  |  |
|  |  | CCDC125 |  |  |  |  |
|  |  | KRTAP29-1 |  |  |  |  |
|  |  | RPS6 |  |  |  |  |
|  |  | PLOD1 |  |  |  |  |
| **Supplementary Table 3. *Continued*** | |  |  |  |  |  |
|  |  | LDLR |  |  |  |  |
|  |  | FAM226B |  |  |  |  |
|  |  | ZMYM4 |  |  |  |  |
|  |  | SPATA3-AS1 |  |  |  |  |
|  |  | MIR1273E |  |  |  |  |
|  |  | LOC100128568 |  |  |  |  |
|  |  | SLC4A10 |  |  |  |  |
|  |  | GNA14 |  |  |  |  |
|  |  | FLNA |  |  |  |  |
|  |  | PDIA4 |  |  |  |  |
|  |  | MBNL2 |  |  |  |  |
|  |  | CLUL1 |  |  |  |  |
|  |  | ZNF207 |  |  |  |  |
|  |  | NAMPT |  |  |  |  |
|  |  | RHPN1-AS1 |  |  |  |  |
|  |  | RAB2A |  |  |  |  |
|  |  | RPL34 |  |  |  |  |
|  |  | GPSM3 |  |  |  |  |
|  |  | ELK3 |  |  |  |  |
|  |  | TRAP1 |  |  |  |  |
|  |  | EMD |  |  |  |  |
|  |  | ZFR2 |  |  |  |  |
|  |  | MTRNR2L3 |  |  |  |  |
|  |  | RPL41 |  |  |  |  |
|  |  | ANAPC15 |  |  |  |  |
|  |  | UBAC2 |  |  |  |  |
|  |  | SPOCK1 |  |  |  |  |
|  |  | ELP3 |  |  |  |  |
|  |  | ZC3H10 |  |  |  |  |
|  |  | SH3RF2 |  |  |  |  |
|  |  | VAC14 |  |  |  |  |
| **Supplementary Table 3. *Continued*** | |  |  |  |  |  |
|  |  | MICAL2 |  |  |  |  |
|  |  | GPR18 |  |  |  |  |
|  |  | RAD21 |  |  |  |  |
|  |  | CTDP1 |  |  |  |  |
|  |  | NCOA2 |  |  |  |  |
|  |  | MIR4779 |  |  |  |  |
|  |  | TMEM245 |  |  |  |  |
|  |  | GREM2 |  |  |  |  |
|  |  | TMUB2 |  |  |  |  |
|  |  | BTBD9 |  |  |  |  |
|  |  | RERE |  |  |  |  |
|  |  | CMTR2 |  |  |  |  |
|  |  | ZNF513 |  |  |  |  |
|  |  | MANBAL |  |  |  |  |
|  |  | PYROXD1 |  |  |  |  |
|  |  | OTUD3 |  |  |  |  |
|  |  | MIR548AN |  |  |  |  |
|  |  | USP12 |  |  |  |  |
|  |  | CSGALNACT1 |  |  |  |  |
|  |  | SRM |  |  |  |  |
|  |  | PCID2 |  |  |  |  |
|  |  | ATP2C1 |  |  |  |  |
|  |  | TIMM23 |  |  |  |  |
|  |  | CDC25B |  |  |  |  |
|  |  | ASB16-AS1 |  |  |  |  |
|  |  | MRPL20 |  |  |  |  |
|  |  | LUCAT1 |  |  |  |  |
|  |  | LOC101927027 |  |  |  |  |
|  |  | AFF1 |  |  |  |  |
|  |  | TPGS1 |  |  |  |  |
|  |  | TMEM30A |  |  |  |  |
| **Supplementary Table 3. *Continued*** | |  |  |  |  |  |
|  |  | MTMR6 |  |  |  |  |
|  |  | KIAA1429 |  |  |  |  |
|  |  | USP10 |  |  |  |  |
|  |  | ICAM2 |  |  |  |  |
|  |  | ASB16 |  |  |  |  |
|  |  | KLHL32 |  |  |  |  |
|  |  | NBPF10 |  |  |  |  |
|  |  | CLEC16A |  |  |  |  |
|  |  | PNP |  |  |  |  |
|  |  | KIAA0355 |  |  |  |  |
|  |  | CD48 |  |  |  |  |
|  |  | PVT1 |  |  |  |  |
|  |  | CCDC88C |  |  |  |  |
|  |  | NOTCH4 |  |  |  |  |
|  |  | STK11IP |  |  |  |  |
|  |  | ATP1B3 |  |  |  |  |
|  |  | ICA1 |  |  |  |  |

| **Supplementary Table 4. Antibodies** | |  |  |  |
| --- | --- | --- | --- | --- |
| Antibody | Clone | Conjugations | Sources | Applications |
| *For Mouse* |  |  |  |  |
| CD45.1 | A20 | FITC | eBioscience | FC |
|  |  | biotin | eBioscience | Cell isolation |
| CD45.2 | 104 | APC | eBioscience | FC |
| CD3 | 17A2 | eFluor450 | eBioscience | FC |
|  |  | none | eBioscience | Stimulation |
| CD4 | RM4-5 | APC-eFluor780 | eBioscience | FC |
| CD8 | 53-6.7 | PE | eBioscience | FC |
|  |  | APC-eFluor780 | eBioscience | FC |
|  |  | V500 | BD Bioscience | FC |
| CD24 | M1/69 | eFluor450 | eBioscience | FC |
| CD25 | PC61 | PE | BD Bioscience | FC |
| CD28 | 37.51 | none | eBioscience | Stimulation |
| CD44 | IM7 | PE-Cy7 | eBioscience | FC |
| CD62L | MEL-14 | APC | eBioscience | FC |
|  |  | APC-eFluor780 | eBioscience | FC |
| CD127 | A7R34 | PE | eBioscience | FC |
|  |  | PE-Cy7 | eBioscience | FC |
| KLRG1 | 2F1 | biotin | eBioscience | FC |
| a4b7 | DATK32 | PE | eBioscience | FC |
| TCRb | H57-597 | biotin | eBioscience | FC |
| IL-2 | JES6-5H4 | PE-Cy7 | eBioscience | FC |
| IFN-g | XMG1.2 | PE | eBioscience | FC |
| TNF-a | MP6-XT22 | APC | eBioscience | FC |
| Foxp3 | MF23 | Alexa647 | BD Bioscience | FC |
|  |  |  |  |  |
| *For Human* |  |  |  |  |
| CD3 | UCHT1 | Pacific Blue | BD Bioscience | FC |
|  | OKT3 | none | eBioscience | Stimulation |
| CD4 | SK3 | APC-H7 | BD Bioscience | FC |
| CD8 | SK1 | APC-Cy7 | BD Bioscience | FC |
| CD28 | CD28.2 | none | eBioscience | Stimulation |
| CD27 | LG.7F9 | FITC | eBioscience | FC |
| CD45 | HI30 | FITC | BD Bioscience | FC |
| CD62L | DREG-56 | APC | BD Bioscience | FC |
| CD127 | HIL-7R-M21 | PE | BD Bioscience | FC |
| CCR7 | 3D12 | PE-Cy7 | BD Bioscience | FC |
| Fas | 7C11 | none | BECKMAN COULTER | Apoptosis induction |
| IL-2 | MQ1-17H12 | APC | BioLegend | FC |
|  |  | FITC | BD Bioscience | FC |
| **Supplementary Table 4. *Continued*** | |  |  |  |
| IFN-g | 4S.B3 | PE | eBioscience | FC |
|  | B27 | PE-Cy7 | BD Bioscience | FC |
|  |  |  |  |  |
| *For ChIP assay* | |  |  |  |
| Histone H3 | D2B12 | none | CST | IP |
| H3K9/14ac | 06-599 | none | Millipore | IP |
| control IgG | #2729 | none | CST | IP |
|  |  |  |  |  |
| *For ChIP-seq* | |  |  |  |
| H3K27me3 | #9733 | none | CST | IP |
| H3K9/14ac | #9677 | none | CST | IP |
| control IgG | #2729 | none | CST | IP |
| FC, Flow Cytometry; CST, Cell Signaling Technology; IP,Immunoprecipitation | | | |  |

| **Supplementary Table 5. Target sequences of shRNA** | | |
| --- | --- | --- |
| Gene symbol | Accession no. | target sequence (5' to 3') |
| RDH10 | NM_172037 | GCACACTTCTGGACCACTAAG |
| NCOR1 | NM_006311 | GCAGAAACACCGCAGTATTGT |
|  |  | GCTCAGGAGGATGAAGATATT |
| SMRT (NCOR2) | NM_006312 | GCAGCCTGGTGCAGATCATCT |
|  |  | GCCCAGCCACTGTCAACAACA |
| Negative control | U47296 | GGTTATGGCAGCACTGCATAA |

| **Supplementary Table 6. Primers for quantitative real-time PCR** | |
| --- | --- |
| Gene symbol | sequence (5' to 3') |
| *For Mouse* |  |
| Rdh10 | Fw: ACACGGGCATGTTCAGAGGCTGC |
|  | Rv: GGGCTGGTCAGTGAGGATGGCCCT |
| Sell | Fw: CCAAGTGTGCTTTCAACTGTTC |
|  | Rv: AAAGGCTCACACTGGACCAC |
| Actb | Fw: TGTCCACCTTCCAGCAGATGT |
|  | Rv: AGCTCAGTAACAGTCCGCCTAGA |
| *For Human* |  |
| RDH10 | Fw: CTGCCGGAGTTGAGGATTAC |
|  | Rv: AGCCTCTGAACATGCCAGTG |
| SELL | Fw: CCCTTTGGGCAAGGACCTGAGACCC |
|  | Rv: AGAGCATTGTCCACCCCCACAACT |
| KLF2 | Fw: CACGCACACAGGTGAGAAGC |
|  | Rv: CACGATCGCACAGATGGCAC |
| NCOR1 | Fw: ACATCAAGACAAACCAGGTGATGA |
|  | Rv: TTTCTCCCATGCCTCCATGAGC |
| NCOR2 (SMRT) | Fw: CAGCAGCGCATCAAGTTCATCA |
|  | Rv: CTCAGCCACTGTCTTCCTCTCC |
| ACTB | Fw: TTGCCGACAGGATGCAGAA |
|  | Rv: GGACAGCGAGGCCAGGAT |
| *For ChIP assay* | |
| CD62L amplicon 1 | Fw: ACGGAGGTGAAGGAACCGAA |
|  | Rv: GCTACCTGTGGTAAATGGCCC |
| CD62L amplicon 2 | Fw: GCACACTCCCTTTGGGCAAG |
|  | Rv: AGGCTCACCATGGCTTTGCT |
| TCF7 Pro | Fw: GGCTAATACGAAGCTGGAAAGTT |
|  | Rv: AGGAACGTCCTCCAGAATAGCT |
| TCF7 Cont | Fw: TGACTTACCACCCAAGTCCCAG |
|  | Rv: CTTCTCCGGGTAAGTACCGAATG |
